# Occurrence of influenza and bacterial infections in cancer patients receiving radiotherapy in Ghana

**DOI:** 10.1371/journal.pone.0271877

**Published:** 2022-07-26

**Authors:** Augustina K. Arjarquah, Evangeline Obodai, Hannah Ayettey Anie, Michael Aning Osei, John Kofi Odoom, Joseph H. K. Bonney, Eric Behene, Erasmus N. Kotey, James Aboagye, Stephen O. Nyarko, Jeannette Bentum, Clara Yeboah, Selassie Kumordjie, Bright Agbodzi, Keren Attiku, Gifty Mawuli, Andrew Letizia, William K. Ampofo, Osbourne Quaye

**Affiliations:** 1 West African Centre for Cell Biology of Infectious Pathogens (WACCBIP), Department of Biochemistry, Cell and Molecular Biology (DBCMB), University of Ghana, Legon, Ghana; 2 College of Health Sciences, University of Ghana-Noguchi Memorial Institute for Medical Research (NMIMR), Legon, Ghana; 3 Cocoa Research Institute of Ghana (CRIG), New Tafo—Akim, Ghana; 4 National Radiotherapy, Oncology and Nuclear Medicine Centre (NRONMC), Korle-Bu Teaching Hospital, Accra, Ghana; 5 United States Naval Medical Research Unit No. 3 (US-NAMRU 3), Ghana Detachment, Accra, Ghana; UNITED STATES

## Abstract

**Background:**

Influenza co-infection with bacteria is a leading cause of influenza-related deaths and severe respiratory infections, especially among high-risk groups like cancer patients undergoing treatment. However, acute respiratory infection (ARI)-like symptoms developed by upper-torso cancer (UTC) patients receiving radiotherapy are considered as side-effects of the radiation. Hence influenza and bacterial pathogens implicated in ARI are not investigated.

**Methods:**

This prospective cohort study examined 85 in-patients with upper-torso cancers undergoing radiotherapy at the National Radiotherapy, Oncology and Nuclear Medicine Centre (NRONMC) of Korle-Bu Teaching Hospital (KBTH) in Accra, Ghana. Eligible patients who consented were recruited into the study from September 2018 to April 2019. Influenza viruses A and B in addition to the following bacteria species *Streptococcus pneumonia*, *Haemophilus influenzae*, *Neisseria meningitidis* and *Staphylococcus aureus* were detected from oropharyngeal and nasopharyngeal swab specimens collected at three different time points. Presence of respiratory pathogens were investigated by influenza virus isolation in cell culture, bacterial culture, polymerase chain reaction (PCR) and next generation sequencing (NGS) assays.

**Results:**

Of the 85 eligible participants enrolled into the study, 87% were females. Participants were 17 to 77 years old, with a median age of 49 years. Most of the participants (88%) enrolled had at least one pathogen present. The most prevalent pathogen was *N*. *meningitidis* (63.4%), followed by *H*. *influenzae* (48.8%), Influenza viruses A and B (32.9%), *S*. *pneumoniae* (32.9%) and *S*. *aureus* (12.2%). Approximately, 65% of these participants developed ARI-like symptoms. Participants with previous episodes of ARI, did not live alone, HNC and total radiation less than 50 Gy were significantly associated with ARI. All treatment forms were also significantly associated with ARI.

**Conclusion:**

Data generated from the study suggests that ARI-like symptoms observed among UTC patients receiving radiotherapy in Ghana, could be due to influenza and bacterial single and co-infections in addition to risk factors and not solely the side-effects of radiation as perceived. These findings will be prime importance for diagnosis, prevention, treatment and control for cancer patients who present with such episodes during treatment.

## Introduction

Acute respiratory infections (ARI) are responsible for about one-third of infectious-related mortality, significant morbidity and a considerable economic burden to health care [1, 2]. These infections are mostly caused by viruses and bacteria that may present as a more severe co-infection. Though ARIs are life threatening and prevalent in children under 5 years, adults above 65 years and in people with immunocompromised systems, they can be self-limiting and of short duration [3, 4]. Symptoms such as sneeze, cough, headache, rhinorrhea, fever ≥ 38°C and sore throat characterize upper respiratory tract infections (URTI) whereas lower respiratory tract infections (LRTI) are characterized by pneumonia, bronchopneumonia, bronchiolitis and bronchitis, lung congestions, difficulty in breathing and loss of consciousness with severe cough [4]. Influenza virus infection remains a primary etiology of ARI globally and its presence increases host susceptibility to bacteria superinfections by affecting the antibacterial innate immune response [5, 6]. The order by which bacterial and influenza infections occur is difficult to differentiate in the clinical setting owing to similar symptomatic presentation. However, when bacterial infections occur after an influenza infection, the morbidity and mortality rate increases [7, 8].

The upper respiratory tract (URT), specifically the nasopharynx, is a niche for microbiome which can become pathogenic and progress into the lower respiratory tract, especially in individuals with impaired immunity causing morbidity and mortality [5, 9]. Impaired innate and adaptive immunity, in addition to mucosal membrane damage and exposure to healthcare environment are factors that contribute to high morbidity and mortality among cancer patients particularly in those with cell-mediated immuno-deficiency [10, 11].

Although newly diagnosed cancer patients prior to any therapy or between therapies have a relatively intact and functional immune system [12, 13], many factors including the type and stage of malignancy, the size and location of tumor, specific deficiencies in host defense mechanisms to cancer, nutritional factors or the model of treatment administered increase the risk of infections among these groups [3, 12]. Additionally, some factors such as intake of alcohol, tobacco smoking, UV exposure and bad diet can be associated with high risk of ARI in patients with cancer [14].

Adjuvant therapies including chemotherapy, radiotherapy, immunotherapy, bone marrow transplant, removal of tumor by surgery, are used for the treatment of most cancers which are known to increase patient’s susceptibility to many types of infections [3, 15–19]. Patients with upper-torso cancers (UTC) including breast and head and neck cancers (HNC) undergoing radiotherapy may present with runny nose, chills, sore throat, cough, mucositis, shortness of breath, fever and lung congestions which are similar to ARI, but may be considered as side effects of the treatment [3, 20, 21]. However, these infections can worsen the patient’s condition and disrupt the treatment process, hence, rapid diagnosis and therapeutic management or intervention is required. In Ghana, the National Radiotherapy, Oncology and Nuclear Medicine Centre (NRONMC) of the Korle-Bu Teaching Hospital (KBTH) estimated that about 50% of 550 patients with UTCs receiving radiotherapy develop ARI-like symptoms each year (personal communication with H. A. Ayittey). The symptoms are perceived as side effects of the radiation, therefore potential respiratory pathogens are not investigated. This study sought to investigate the occurrence of influenza and bacterial infections in patients with upper thoracic cancers receiving radiotherapy in Ghana to determine the prevalence of infectious etiologies among participants who present ARI-like symptoms.

## Methods

### Patient enrollment

Upper-Torso Cancer (UTC) patients with breast and head and neck cancers undergoing radiotherapy but asymptomatic for ARI before treatment were enrolled into a prospective hospital-based dynamic case-cohort study from September 2018 to May 2019.

Preceding enrollment, eligible patients who met the recruitment criteria gave a written informed consent after a thorough and careful explanation of the purpose and procedures of the study in English, French or Twi. Participants were recruited by trained radiotherapist from NRONMC, KBTH and myself, a senior research assistant with the National Influenza Center, Ghana. A well-structured questionnaire was used to obtain demographic, clinical, and risk factor data S1 Fig. An eligible patient was defined as an UTC patient yet to undergo radiotherapy and asymptomatic for ARI prior to the start of treatment. On the contrary, a UTC patient symptomatic for ARI prior to treatment and those receiving less than five (5) fractions of prescribed dosage were excluded from this study. The study was conducted at the NRONMC of the Korle-Bu Teaching Hospital (KBTH) sited in Accra, Ghana, a tertiary and leading national medical referral Centre for Ghana, which also draws a number of their clientele form other African sub-regions. Ethical clearance was obtained from the Institutional Review Boards of Noguchi Memorial Institute for Medical Research (NMIMR-IRB CPN 091/17-18) and KBTH (KBTH-IRB/000116/2018) before the start of the study S2 Fig.

### Sample collection

Oropharyngeal and nasopharyngeal swab specimens were collected from each participant at three different time points using a sterile flexible nylon tip flocked swab (Copan Flocked Technologies, Italy). Samples were collected same day before participants began treatment, half way through treatment irrespective of whether participants were symptomatic or asymptomatic for ARI, and on the last day of treatment, where a complete dosage of radiation had been taken. Treatment sessions of participants were within two to six weeks. The nasopharyngeal and oropharyngeal swab specimens collected at each time point were stored in 2 ml viral transport medium (VTM) and 0.01% phosphate buffered saline (PBS) at -80°C prior to isolation of influenza virus RNA and bacterial DNA respectively. The first sample taken prior to start of treatment served as the baseline or control for each participant, which was compared with latter specimens.

### Cell culture and influenza virus isolation

All samples collected in VTM were cultured on 80%-90% confluent Madin-Darby Canine Kidney (MDCK) cell line in order to attempt virus isolation by hemagglutination (HA) assay before detection by real-time RT-PCR. This was to increase the viral load for further analysis. The cell, FR-58-MDCK, London Line was obtained from the International Reagent Resource (IRR), formerly the Influenza Reagent Resource (IRR) established by the Centers for Disease Control and Prevention (CDC). Cultured samples were observed each day for cytopathic effect (CPE) and contamination.

#### Identification and detection of respiratory pathogens by Real-Time/ RT-PCR

Specific primers targeting specific genes of interest were used for the detection of respiratory pathogens (Table 1). Influenza virus was detected with SuperScript III Platinum One-Step quantitative RT-PCR kit (Invitrogen, USA) using protocols defined by the United States Centres for Disease Control and Prevention [22] with slight modifications to the manufacturer’s pipetting protocol. Likewise, for the detection of *H*. *influenzae*, *S*. *pneumoniae* and *N*. *meningitidis*, protocol used by Thomas et al. 2011 [24] was slightly modified and optimized. Detection was by real-time PCR using Power SYBR^®^ Green PCR Master Mix Kit (Applied Biosystems, Carlsbad, California, USA) in StepOne ABI 7500 PCR System (Applied Biosystems, Carlsbad, USA). Reaction mixtures for detection of *H*. *influenzae*, *S*. *pneumoniae* and *N*. *meningitidis* contained 7.04 μL of nuclease free water, 10.04 μL 2 x RT-PCR reaction mix, 0.16 μL of RT-Enzyme mix, 0.4 μL of forward primer, 0.8 μL and 2 μL template DNA. Amplification of template were 2 minutes at 50°C reverse transcription stage, 10 minutes at 95°C to activate the Taq polymerase enzyme, and 45 cycles of 15 seconds at 95°C and 60 seconds at 60°C for final elongation. At each run, a melting or dissociation curve analysis was done to confirm the amplification curves of our target.

**Table 1:** Oligonucleotides sequences for PCR.

Name	Polarity	Oligo sequence (5’→3’)	Gene	*p*M
**Influenza A**				
GRAM/7F	Forward	CTTCTAACCGAGGTCGAAACGTA		100
GRAM/161R	Reverse	GGTGACAGGATTGGTCTTGTCTTTA		100
GRAM probe/52/+	Probe	FAM–TCAGGCCCCCTCAAAGCCGAG-3’-BHQ-1		100
**A(H3N2)**				
H3h-319F	Forward	AGCAAAGCCTACAGCAA	HA	200
H3h-377R	Reverse	GACCTAAGGGAGGCATAA	HA	200
H3h-358 Probe	Probe	FAM-CCGGCACATCATAAGGGTAACA3’-BHQ-1	HA	200
**A(H1N1) *pdm09***				
GRswH1-349F	Forward	GAGCTAAGAGAGCAATTGA	HA	200
GRswH1-601R	Reverse	GTAGATGGATGGTGAATG	HA	200
GRswH1-538 Probe (-)	Probe	FAM-TTGCTGAGCTTTGGGTATGA 3’-BHQ-1	HA	200
**Influenza B**				
HA(B)-1102F	Forward	ATTGCTGGTTTCTTAGAAGG	HA	200
HA(B)-1226R	Reverse	TTGTTTATRGCTTCTTGMGT	HA	200
HA(B)-1125probe (+)	Probe	FAM-ATGGGAAGGAATGATTGCAGGT-BHQ1	HA	200
***N*. *meningitidis***				
F351	Forward	GCACACTTAGGTGATTTACCTGCAT	*sodC*	196
R478	Reverse	CCACCCGTGTGGATCATAATAGA	*sodC*	391
***H*. *influenzae***				
HelS-F	Forward	CCGGGTGCGGTAGAATTTAATAA	*Hel S*	196
HelA-R	Reverse	CTGATTTTTCAGTGCTGTCTTTGC	*Hel S*	391
***S*. *pneumoniae***				
F373	Forward	ACGCAATCTAGCAGATGAAGCA		196
R424	Reverse	TCGTGCGTTTTAATTCCAGCT	*Lyt A*	391
***S*. *aureus***				
*mec*AP4	Forward	TCCAGATTACAACTTCACCAGG	*mecA*	
*mec*AP7	reverse	CCACTTCATATCTTGTAACG		
*spa*-1113f	Forward	TAAAGACGATCCTTCGGTGAGC	*spa*	
*spa*-1514	reverse	CAGCAGTAGTGCCGTTTGCTT		
*pvl*-FP	Forward	GCTGGACAAAACTTCTTGGAATAT	*pvl*	
*pvl*-RP	reverse	GATAGGACACCAATAAATTCTGGATTG		

Abbreviations: pM = picomoles: final concentration in PCR reaction.

Fluorescence dyes: FAM = 6-Carboxyfluorescein; BHQ = Black Hole Quencher.

Viral protein gene: HA = Hemagglutinin; Bacterial Protein genes: *SodC* = Superoxide dismutase [Cu-Zn];

*Hel S* = Lipoprotein E; *Lyt A* = Autolysin A; *pvl* = Panton Valentine leucocidin, *spa = staphylococcal* protein A, *mecA =* Methicillin resistance gene.

All oligonucleotides for virus and bacteria detection were purchased from Eurofins MWG Synthesis GmbH (Germany) and Inqaba Biotech West Africa Ltd., Ghana Branch (Ghana) respectively.

### Detection of *S*. *aureus* by conventional PCR

For the detection of *S*. *aureus*, a working primer mix targeting the *mecA* (0.45 μM), *pvl* (1 μM) and *spa* (0.18 μM) genes was prepared [23]. DNA extracted from each sample was amplified by conventional PCR and products were run on a 2% agarose gel by electrophoresis. The expected band sizes for the various genes were: 162 bp (*mecA*), 80 bp (*pvl*) and 200 bp—600 bp (variable region of *spa*) [23]. The amplicons were visualized and analyzed using the BioDocAnalyzer instrument and software.

### Next generation sequencing of *S*. *aureus*

By using a Qubit 4 Fluorometer (ThermoFisher Scientific, Singapore), genomic DNA isolated from *S*. *aureus* positive samples were accurately quantified. Nextera DNA Flex Pre-Enrichment Library Prep and Enrichment Reagents (Cat # 20025524) were used for generating the DNA libraries. The concentration of the DNA was normalized to have an input of 100 ng-500 ng before tagmentation. The gDNA were tagged with Bead-Linked Transposomes (BLT) to aid in the fragmentation of the DNA before the addition of adapter sequences for easy identification. A post tagmentation cleanup was done after the addition of a tagment stop buffer (TSB) to wash off any unbound DNA to the BLT before PCR amplification. Nextera DNA Flex-specific index adapter sequences were added to the ends of tagmented DNA fragments and limited-cycle PCR was run to amplify the tagmented DNA. The libraries created after amplification were thoroughly cleaned through a double-sided bead purification procedure. The integrity and quality of the library prepared were checked and analyzed using an Agilent 2100 Bioanalyzer with a high sensitivity DNA kit. The libraries were quantified by qPCR using the ABI 7300/7500 Real-Time PCR system (Applied Biosystems, USA). DNA libraries obtained were pooled together, normalized and all double-stranded DNA were denatured into single strands using NaOH. The pooled libraries were finally loaded into the Illumina Miseq Sequencer for sequencing.

### Statistical analysis

Descriptive statistics were used to describe the overall study population of UTC patients using SPSS Version 22 (SPSS Inc., Illinois, U.S.A). Pearson Chi-square test, correlation and other measures were used to determine the association between ARI during treatment and dosage of radiation, type of cancer and intent and form of treatment. Data for these analyses were extracted from a well-structured questionnaire administered to all participants. P-values were two-sided and considered significant at a level ≤ 0.05. Association of respiratory pathogens to clinical presentations were analyzed using odds ratio at 95% confidence interval (CI).

## Results

### Demographics and clinical characteristics

A total of 85 participants were enrolled into the study. The median age of the participants was 49 years with the youngest being 17 years. Participants within the age bracket of 51years to 60 years were the most frequently (30%) enrolled. The cohort consisted mostly of females (88%); majority (92%) were Ghanaians and the rest were from other West African countries. Breast cancer (80%) was the most prevalent malignancy recorded. In addition, 83.5% of all participants received curative care with the remaining (16.5%) put on palliative care (p = 0.001) (Table 2). Most of the participants (65%) had earlier undergone either surgery or chemotherapy prior to receiving radiotherapy as the adjuvant. Also, 26% of participants received radiotherapy as the primary treatment, while about 9% of participants received chemotherapy and radiotherapy concurrently. Three of the participants were lost to follow up during the study.

**Table 2:** Demographics and clinical characteristics of study participant.

Characteristics	N (%)	Pathogen Positivity	Incidence of ARI	*p*-values
**Total Population**	**85 (100)**	**72 (87.8)**	**53 (64.6)**	
**Age groups**				
<20	2 (2.4)	1 (1)	1 (2)	1
21–30	4 (4.7)	4 (6)	2 (4)	0.361
31–40	19 (22.4)	17 (24)	13 (25)	0.129
41–50	21 (24.7)	15 (21)	12 (23)	0.529
51–60	26 (30.5)	24 (33)	15 (28)	0.062
>60	13 (15.3)	11 (15)	10 (19)	0.255
**Gender**				
Female	74 (87.1)	63 (88)	45 (85)	
Male	11 (12.9)	9 (12)	8 (15)	0.776
**Nationality**				
Beninoire	1 (1.2)	1 (1)	1 (2)	1
Ghanaian	79 (91.8)	68 (93)	51 (94)	0.687
Liberian	2 (2.4)	2 (3)	1 (2)	0.504
Sierra Leonean	2 (2.4)	0 (0)	0 (0)	0.083
Togolese	2 (2.4)	2 (3)	1 (2)	0.504
**Types of cancer**				
Head and Neck	18 (21.2)	13 (18)	12 (23)	
Breast Cancer	67 (78.8)	59 (82)	41 (77)	0.097
**Form of treatment**				
Curative care	71 (83.5)	64 (89)	46 (87)	
Palliative care	14 (16.5)	8 (11)	7 (13)	0.001*

Head and Neck Cancers: Maxillary, Nasopharyngeal, Laryngeal, Myoepithelial, Conjunctiva, Medulloblastoma and Parotid cancers; Palliative Care: Treatment to ease cancer symptoms and improve the quality of life of patients; Curative care: Treatment with intention of curing the cancer; N = Study population size; N = 85. **However, 3 were lost to follow-up.**

#### Incidence of ARI-like symptoms among participants after exposure to radiation

**Fig 1:**
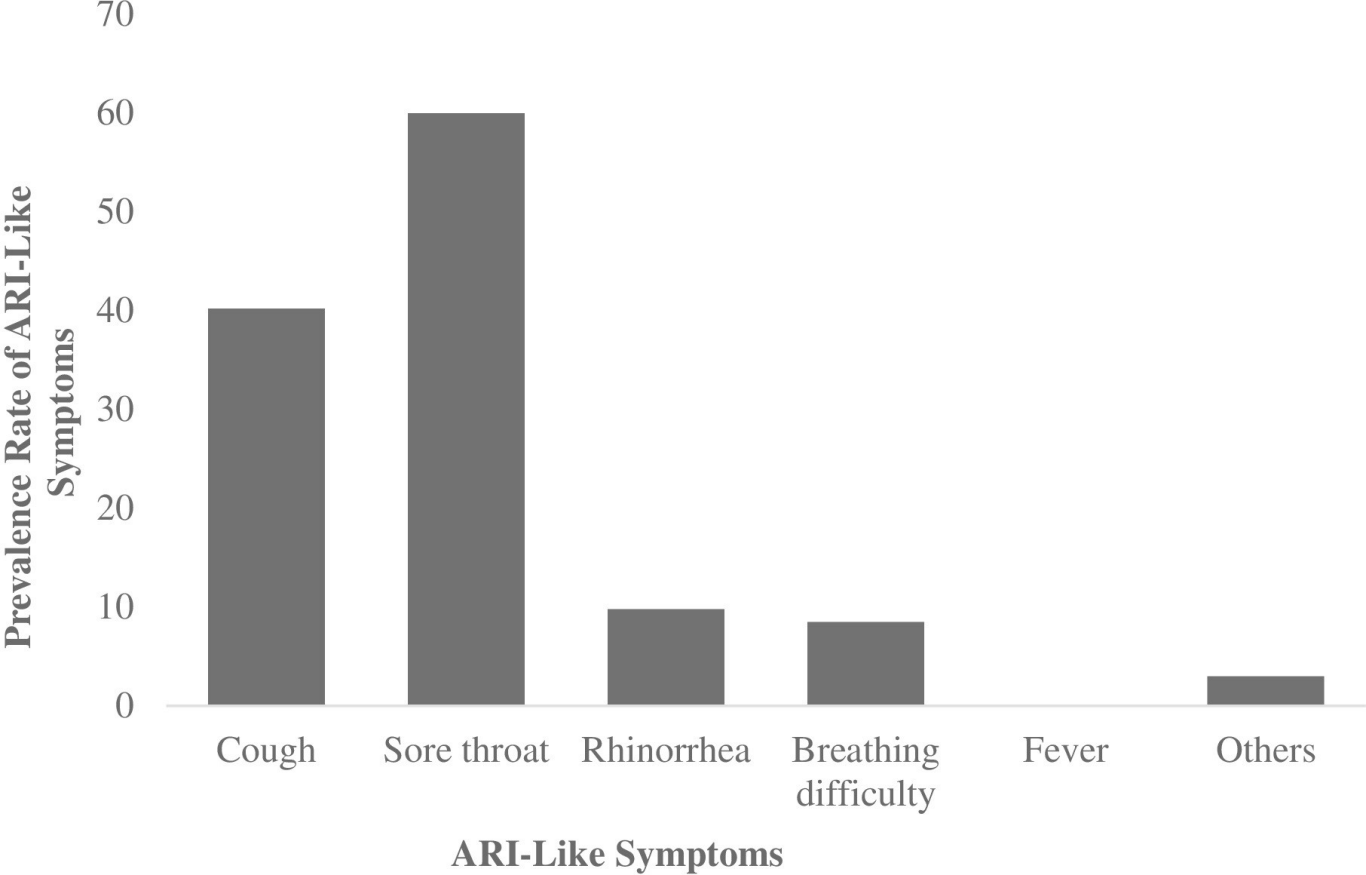
Prevalence of ARI-like symptoms detected among cohort after radiation exposure. Study population size (82).

Most of the participants (74%) presented with at least one of these ARI-like symptoms: Sore throat (60%), cough (40.2%), rhinorrhea (9.8%) and breathing difficulty (8.5%) during treatment. About 3% of the cohort complained of mucositis, fatigue, odynophagia and anorexia during radiation exposure (Fig 1). However, none of the participants reported or came up with fever during the study.

### Prevalence of influenza virus and bacterial pathogens

**Fig 2:**
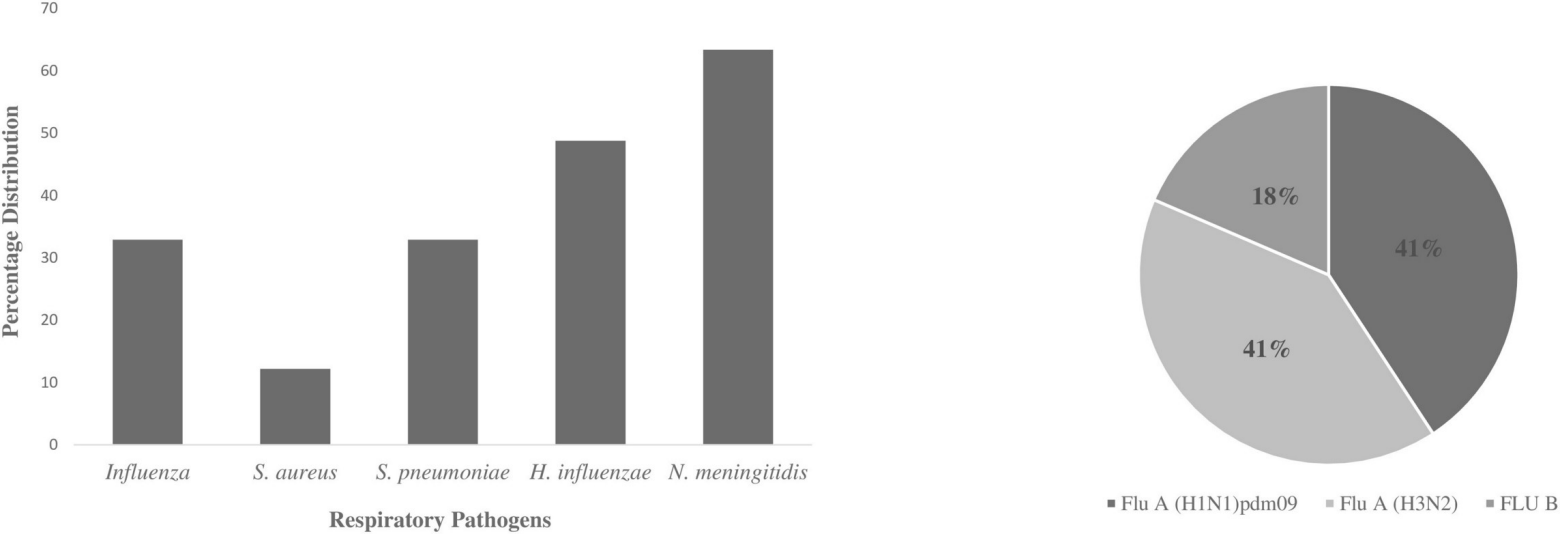
**a.** Prevalence of respiratory pathogens detected among the cohort. Study population size (82); **Influenza positive samples** were cultured on MDCK cell line for virus isolation before molecular detection by Real-Time RT PCR. **b.** Percentage distribution of Influenza virus types detected among the cohort. The presence of single and multiple pathogens were evaluated among the cohort during treatment (Fig 3A). Of the 82 eligible participants, single pathogens were detected in 21(25.61%) of cohort and multiple pathogens in exactly 50% (41) of the cohort. The multiple infections included 22 (26.83%) double, 14 (17.07%) triple and 5 (6.10%) quadruple infections. About 24.39% (20) of the cohort had no pathogen present during the study even though some presented with ARI-like symptoms during treatment (Fig 3A). *H*. *influenzae* and *N*. *meningitidis* were more commonly identified with all other pathogens (Table 3). Influenza and bacterial co-infection was also observed among 34.15% of the cohort during treatment (Fig 3B), a combination of influenza and at least one of the bacterial pathogens with the exception of *S*. *aureus*.

A respiratory pathogen was detected in 89% of the analyzed cases. Cumulatively, *N*. *meningitidis* (63.4%) was the most prevalent pathogen detected followed by *H*. *influenzae* (48.8%), Influenza virus (32.9%), *S*. *pneumoniae* (32.9%) and *S*. *aureus* (12.2%) (Fig 2A). About 18% of the participants were positive for Influenza B virus. Influenza A virus typing revealed equal prevalence of sub-types A-H3N2 (41%) and A-H1N1*pdm09* (41%) as shown in Fig 2B.

**Fig 3:**
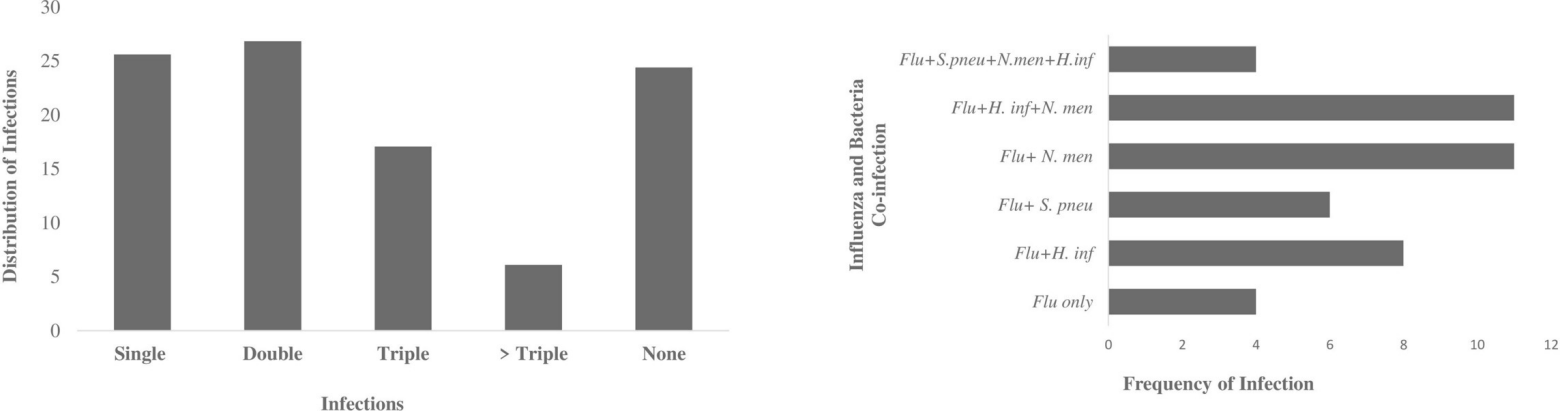
**a.** Single and Multiple pathogen detections among cohort during treatment. Study population size (82). **b.** Frequency of influenza and bacteria Co-infection among cohort with ARI.

**Table 3:** Influenza and bacterial infections.

Pathogen type	Influenza A	Influenza B	*S*. *pneumoniae*	*S*. *aureus*	*H*. *influenzae*	*N*. *meningitidis*
**Influenza A**	**6**	0	6	0	6	8
**Influenza B**		**0**	0	0	2	3
***S*. *pneumoniae***			**3**	4	10	10
***S*. *aureus***				**0**	4	4
***H*. *influenzae***					**3**	25
***N*. *meningitidis***						**10**

All *S*. *aureus* isolates were positive for the virulent *spa* gene with only one carrying the *Panton-Valentine leucocidin* (PVL) gene. None of the isolates had the *mecA* gene. Of the 10 (12.2%) *S*. *aureus* positive samples, 4 (40%) were successfully sequenced. These isolates were characterized by *spa* typing, multi-locus sequence typing and the presence of other exo-toxin genes using the Centre for Genomic Epidemiology (CGE) bioinformatic tool. The *spa* types of 3 of the successfully sequenced isolates were unknown, however, one of the isolates was identified as t334 type with *spa* repeat 11-12-21-17-34-22-25 S1 Table. All 4 sequences generated in this study have been deposited into the GenBank with the following accession numbers: CP096248, CP096249, CP096250 and CP096251.

### Presence or absence of pathogens before and after radiation exposure

**Fig 4:**
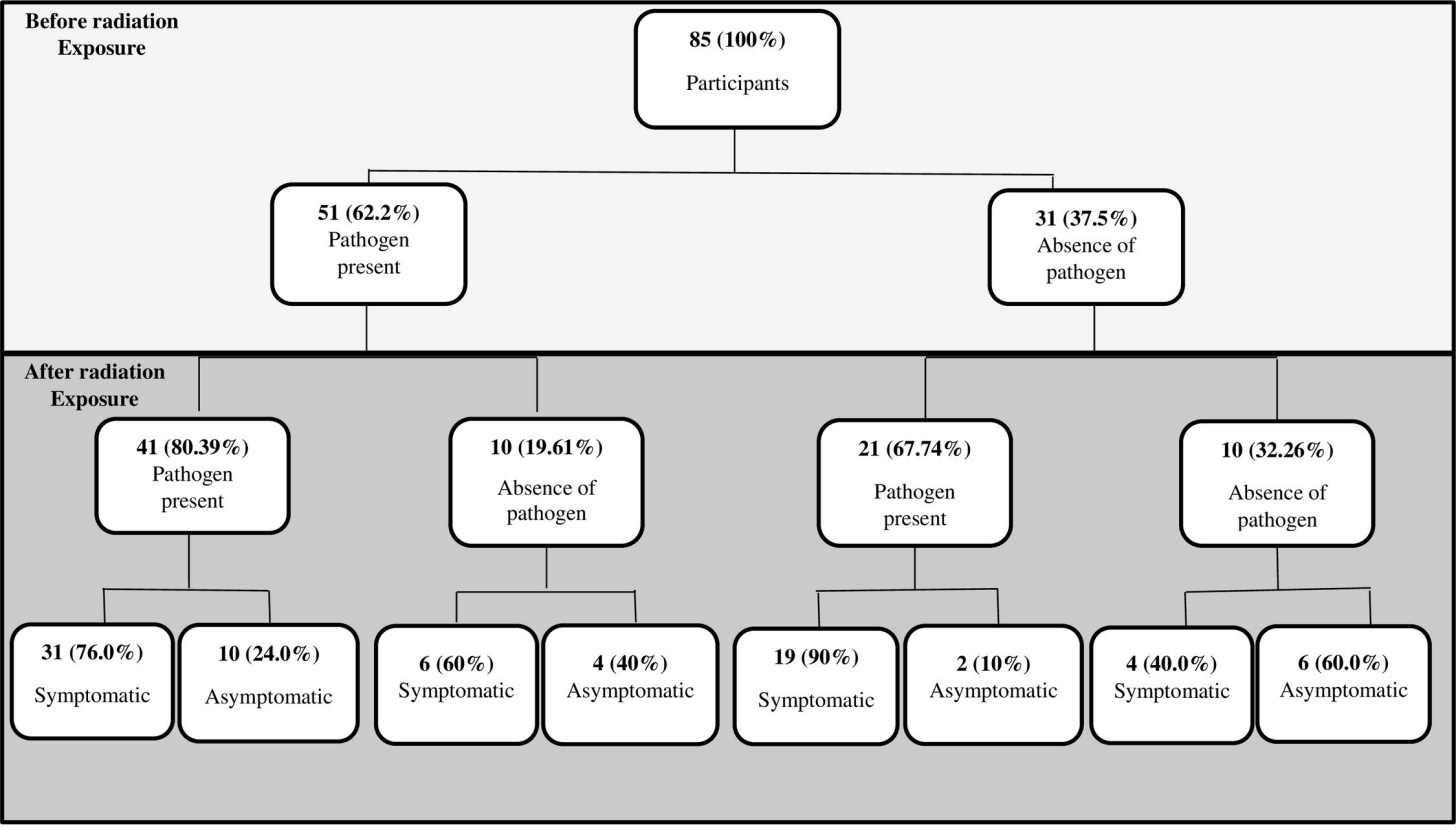
Presence or absence of respiratory pathogens before and after radiation exposure among cohort. Study population size (82); the 3 lost to follow up were excluded.

During the study, four main observations were made in relation to the presence or absence of a pathogen either before or during treatment. About 62.2% (51) of the eligible participants had at least one pathogen before exposure to radiation (Fig 4). Out of this group, 80.39% (41) had the pathogens persist after exposure to radiation with approximately 76% (31) developing ARI-like symptoms. Although 19.61% (10) of this group had no pathogen present during treatment, 60% (6) developed ARI-like symptoms. It was also observed that, 67.74% (21) of participants who had no pathogen present before start of treatment developed ARI after exposure to radiation, and about 90% (19) presented with ARI-like symptoms. Only 32.26% (10) of the study participants had no pathogen present throughout the study, nevertheless, 40% (4) of this group presented with ARI-like symptoms.

### Association of respiratory pathogens with ARI symptoms

Fishers exact test was used to determine any association between ARI-like symptoms and pathogens detected. Influenza virus and *H*. *influenzae* were associated with at least one of the clinical symptoms although association was statistically not significant. However, *S*. *aureus* infections was found to increase the risk of cough (OR 7.17; 95% CI = 2.38 to 21.61), rhinorrhea (OR 3.83; 95% CI = 1.63 to 9.0) and difficulty in breathing (OR 3.68; 95% CI = 1.68 to 8.05) whereas *N*. *meningitidis* and *S*. *pneumoniae* had an association with only rhinorrhea at (OR 3.12; 95% CI = 1.72 to 5.68) and (OR 2.45; 95% CI = 1.37 to 4.39) respectively as shown in Table 4.

**Table 4:** Association of respiratory pathogens with ARI-like symptoms. OR (95% CI).

Symptoms/ Pathogen	Influenza Virus	*S*. *pneumoniae*	*S*. *aureus*	*H*. *influenzae*	*N*. *meningitidis*
**Cough**	1.25 (0.70–2.24)	0.92 (0.51–1.64)	**7.17 (2.38–21.61)**	1.43 (0.82–2.51)	1.41 (0.31–0.24)
**Rhinorrhea**	1.36 (0.76–2.45)	**2.45 (1.37–4.39)**	**3.83 (1.63–9.00)**	1.13 (0.65–1.96)	**3.12 (1.72–5.68)**
**Sore Throat**	1.21 (0.6–2.20)	1.58 (0.871–2.89)	1.00 (0.43–2.35)	1.00 (0.57–1.74)	1.18 (0.67–2.09)
**Difficulty Breathing**	0.73 (0.40–1.32)	1.18 (0.67–2.07)	**3.68 (1.68–8.05)**	0.85 (0.49–1.49)	1.63 (0.91–2.94)

Study population size (82)

#### Association of ARI with cancer types, treatment options, radiation dosage and other pre-disposing factors

Malignancy type, treatment options, total radiation dosage administered and pre-disposing factors such as previous episodes of ARI, contact with an ARI infected person, participants who did not live alone, antibiotic intake, behavioral conditions and co-morbidities were analyzed for association with ARI. About 21 (67.7%) of the 31 participants who had no infections before the start of treatment but developed ARI during treatment were considered (Table 5). From the results, there was a signficant association of ARI with participants who presented with HNC, total radiation less than 50 Gy and all treatment forms at p < 0.05. There was however no significant association of ARI with patients who received palliative care.

**Table 5:** Association of ARI with cancer type, treatment options and dosage of radiation.

Clinical Parameters		Patients with ARI	OR (95% CI)	*p-value*
		n (%)		
**Cancer Types:**				
Head and Neck (HNC)	n = 11	9 (81.8)	3.04 (1.59–5.81)	< 0.001*
Thoracic	n = 20	12 (60.0)	1	
**Intent of treatment**:				
Palliative care	n = 8	6 (75.0)	1.62 (0.88–2.98)	0.082
Curative	n = 23	15 (65.2)	1	
**Form of Treatment (Setting of Radiotherapy):**				
Primary treatment	n = 10	9 (90)	6.52 (3.20–13.27)	<0.0001*
Adjuvant	n = 16	9 (56.3)	0.33 (0.18–0.61)	0.001*
Concomitant	n = 5	3 (60.0)	0.55 (0.33–0.92)	0.016*
**Radiation dosage:**				
< 50 (Gy)	n = 9	7 (77.8)	1.99 (1.07–3.73)	0.02* 1
≥ 50 (Gy)	n = 22	14 (63.6)	1	

* = p-value <0.05; N = subgroup of patients who developed ARI during treatment only; Population size (31), n = Frequency of patients presenting with ARI; ARI = patients with at least one respiratory pathogen and clinical symptoms

Also, participants who had had previous episode of ARI before the start of treatment, those who did not live alone, and those who had co-morbidities such as hypertension and diabetes had a significant association with ARI (p < 0.05) (Table 6).

**Table 6:** Pre-disposing factors associated with ARI among the cohort.

Pre-disposing factors	No. of patients with risk factor, n (%)	ARI n (%)	OR (95% CI)	*p-value*
Previous episode of ARI	8 (9.8)	4 (50.0)	4.00 (1.88–8.45)	<0.001*
Contact with ARI infected persons	9 (11.0)	5 (55.6)	0.63 (0.35–1.11)	0.146
***Household size*:**				
Lives alone	14 (17.1)	7 (50.0)	1	
Does not live alone	68 (82.9)	46 (67.7)	2.13 (1.20–3.78)	0.014*
***Behavioral Conditions*:**				
Smoking	3 (3.7)	2 (66.7)	1.09 (0.61–1.96)	0.881
Alcohol	20 (24.4)	14 (70.0)	1.26 (0.69–2.27)	0.546
Passive smoking	22 (26.8)	14 (63.6)	1.00 (0.53–1.71)	1.000
Antibiotic intatke	3 (3.7)	2 (66.7)	1.09 (0.61–1.96)	0.881
***Other co-morbidities*:**				
Diabetes	9 (10.8)	8 (88.9)	5.00 (2.35–10.45)	<0.0001*
Hypertension	28 (34.1)	22 (78.6)	2.84 (1.52–5.29)	0.001*

* = p-value <0.05; N = population size; N = 82; **Reference groups:** Number of persons in household: Persons living alone and behavioral conditions and other co-morbidities: Patients negative for behavioral responses or other co-morbidities

## Discussion

Acute respiratory infection (ARI) is known to cause significant morbidity and mortality among high-risk groups, particularly the immunocompromised [10]. Although studies have demonstrated the importance of ARI in cancer patients, the actual role of these infections remains unclear [24], especially in radiotherapy, since ARI-like symptoms developed by patients with UTC during treatment are often considered as side effects of the radiation [20, 21].

In this study, ARI was defined as presence of at least one pathogen with participant presenting with one or more ARI-like symptoms. Cough and sore throat were the most common symptoms developed during treatment, followed by rhinorrhea and breathing difficulty. None of the participants reported or presented with fever during the study. These observations are similar to findings in other cancer studies [10, 16, 25, 26]. Studies by Sickles *et al*. and Strojnik *et al*. also demonstrated that fever may be absent in some cancer patients regardless of microbiologically and/or clinically confirmed infections [27, 28].

Influenza virus types A and B, *S*. *pneumoniae*, *H*. *influenzae*, *S*. *aureus* and *N*. *meningitidis* were the main pathogens identified by PCR. There was a relatively high prevalence of respiratory pathogens and high incidence of ARI. The univariate analysis conducted indicated that, the presence of a pathogen correlated well with at least one of the ARI-like symptoms which is similar to other studies from different countries [3, 24, 28, 29]. Additionally, the high prevalence (61%) of bacteria in this study confirms the findings of Margolis and colleagues [30] which suggests that, these pathogens interact with one another in a common niche, hence may co-colonize. The high prevalence (63.4%) of *N*. *meningitidis*, *H*. *influenzae* (48.8%), *S*. *pneumoniae* (32.9%) and *S*. *aureus* (12.2%) supports the interaction of these pathogens with each other in a common niche [29, 31], although *N*. *meningitidis* is an uncommon cause of respiratory infections [32]. The presence of a virus is speculated to predispose bacterial colonization through enormous but variable mechanisms. Influenza and bacterial co-infection observed among the cohort during treatment could be through enormous and variable mechanisms including epithelium barrier disruption and production of viral factors that influence the interactions as established in other studies [5, 33–36].

Methicillin-resistant and virulent *S*. *aureus* has been reported to be prevalent in wound infections, however, the production of *Panton-Valentine leucocidin* (*pvl*) toxin, Protein A (encodes spa gene) and hemolysin by the bacteria can cause respiratory infections including pneumonia [33]. All isolates were positive for the virulent *spa* gene which has currently been observed to be involved in the pathogenesis of *S*. *aureus* pneumonia [37]. Though none of the isolates carried the *mecA* gene associated with hospital-acquired methicillin resistant *S*. *aureus* (HA-MRSA), one of the isolates had the *pvl* gene associated with community-acquired methicillin resistant *S*. *aureus* (CA-MRSA). Further characterization of these isolates by whole genome sequencing identified one of the isolates as a t334 *spa*-Type which is inconsistent with prevalent *spa*-Types t355, t084 and t008 circulating in Ghana [5, 33, 38]. Interestingly, 3 out of 4 successfully sequenced isolates were non-*spa*-Typeable which could be considered as novel clones of *S*. *aureus* as seen in a recent study in Germany [39].

The presence or absence of a pathogen observed before or during treatment was well evaluated and were categorized in to four groups. The first group encompassed participants who had at least one pathogen present prior to treatment that persisted during treatment, and also developed ARI-like symptoms after exposure to radiation. The presence of a pathogen before treatment could be a result of a compromised immune system due to the cancer [3], whereas the persistence of these pathogens accompanied with ARI-like symptoms could be due to a more weakened immune system through the destruction of immune cells by the radiation [12, 40]. Another group had pathogens present before treatment, nevertheless, there was no infection after exposure to radiation despite developing ARI-like symptoms. The absence of pathogens in this group could be associated with the inactivation of these pathogens by the radiation during treatment [41–43]. The ARI-like symptoms developed by this group could possibly be the side effects of radiation [20, 21]. The third group were those who had no pathogens present prior to treatment but later developed ARI-like symptoms during treatment, which could be a result of destruction of immune cells by radiation hence making participants susceptible to infections [12, 40], or nosocomial infection contracted from other group of participants who had the pathogen present during the study. The last group were those who had no pathogens present during the study, however developed ARI-like symptoms, which could be attributed to the radiation administered [20, 21].

Results from this current study also indicate a significant association of ARI with HNC and this could be due to the location of the cancer since HNC mostly affect the upper airways serving as an effective barrier to infection [44–46]. Also, most of the HNC patients reported with advanced cancers at clinical stages III and IV, putting them at increased risk of a more compromised immune system hence susceptible to infections including ARI [47, 48]. Our findings also illustrated a significant association of ARI with all treatment modalities administered to participants during the study which is consistent with various studies [12, 40, 41]. One will only be given palliative care when the cancer is extremely advanced and incurable. The advaced stage impairs their immunity to multiple etiologies, hence increasing susceptibility to infections including ARI [48, 49]. Results from this study was however contradictory to the above since there was no significant association of palliative care with ARI.

Currently, opinions about the ionizing radiation interaction with the immune system are conflicting [50–54]. Previously, high dose ionizing radiation was considered as the major cause of immune suppression in cancer patients due to the radio-sensitivity of the lymphoid system [50–52]. This could mean that patients who received higher doses of radiation, could be more prone to infections due to the death of immune cells. However, presently, the effect of radiation on the immune system and microenvironment of the tumor has been improved by modifications in radiation dose fraction regimens (standard fraction of 2 Gy) and the dose delivery methods [50, 51, 53]. We therefore infer that the significant association of ARI to radiation dosage less than 50 Gy could be a result of other external factors that pre-disposed the cohort to ARI. Nevertheless, different hypothesis about specific effect of different dosage and fractionation on the anti-tumoral response and immune system are under investigation [50].

Cancer increases the risk of infection; however, some environmental, medical and behavioral factors predispose non-immunocompetent individuals to ARI. Most studies have reported gender, age, nutritional factors, exposure to infected persons, household size, parental or passive smoking to be associated with ARI. Majority of these findings have been more specific to children under five years [4, 54, 55]. Our study found that, previous episode of ARI, household size and co-morbidities like diabetes and hypertension were strongly associated with ARI. This is similar to observations made in an Australian community on risk factors associated with ARI with the exception of contact or exposure with infected person which was contrary to their findings [56]. Although other studies reported significant association of ARI with behavioral conditions like smoking, alcohol intake and passive smoke inhalation or parental smoking, the results from this study was inconsistent with those findings [54–56].

## Conclusion

The findings from this study are the first of its kind in Ghana from reviewed literature and information from the NRONMC, KBTH. This study demonstrated a high burden of ARI due to the presence of influenza virus, *S*. *pneumoniae*, *N*. *meningitidis*, *H*. *influenzae* and *S*. *aureus*. Novel virulent *S*. *aureus* strains were detected among the cohort. There was a high incidence of co-infections among the respiratory pathogens detected, which could be an indicative factor for ARI among the cohort. Stage and type of malignancy, the intent and forms of treatment, total dosage of radiation administered to participants and risk factors such as household size, a previous episode of ARI and other co-morbidities were associated with ARI among the cohort. The study also demonstrates that ARI-like symptoms developed by UTC patients receiving radiotherapy at the KBTH in Accra, Ghana could be due to the presence of community respiratory pathogens; and risk factors associated with ARI but not solely the side effects of the radiation as perceived. The findings can be useful in the provision of guidelines needed for effective prevention and management of ARI in UTC patients receiving radiotherapy. We recommend routine screening and testing of ARI among UTC patients receiving radiotherapy and also prophylactic treatment with antiviral and antibiotics to patients who present with ARI during treatment.

## Supporting information

S1 Fig Enrolment form/questionnaire. (PDF)

S2 Fig Ethical clearance. (PDF)

S1 Table Characterization of S. aureus by spa type, MLST and exo-toxin genes. MLST = Multi-locus sequence type; hlgA = gamma-hemolysin chain II precursor, hlgB = gamma-hemolysin component B precursor, hlgC = gamma-hemolysin component C, luk = leucocidin, luk-PV = Panton Valentine leucocidin; * = nearest hit.
(DOCX)

S1 File Minimal data. (XLSX)
