# Supplementary material for: Occurrence of influenza and bacterial infections in cancer patients receiving radiotherapy in Ghana
Source: PLoS One. 2022 Jul 26;17(7):e0271877. doi: 10.1371/journal.pone.0271877 (PMC9321433; doi:10.1371/journal.pone.0271877)
Supplement: S1 Fig — (PDF) [file pone.0271877.s001.pdf]

# ENROLLMENT FORM/ QUESTIONAIRE

Date of Enrollment: ……. /…../……..

## PART I

### Demographic Details

1. Participant ID: …………………………...
2. Place of residence: ………………………
3. Age: ……………………………………..
4. Sex: Male ☐ Female ☐
5. Nationality: …………………..................
6. Occupation: ……………………………...

### Patient Clinical History

7. Diagnosis: …………………………………………
8. Cancer Stage……………...
9. Intent of treatment: Radical ☐ Pre-Op/Post-Op ☐ Palliation ☐

**Chemotherapy**

10. Type of Chemotherapy: ……………………………………………………………….
11. Intent of Chemotherapy: Adjuvant ☐ Neoadjuvant ☐ Concomitant ☐
12. Number of cycles……………………….
13. Date treatment started...…………………
14. Last day of treatment……………………
15. Mode of administration…………………

**Radiotherapy**

16. Fractionation…………………………….
17. Dosage of radiation: …………………….
18. Date treatment started...…………………
19. Duration of treatment: …………………..

## PART II

**Exposure to Risk Factors**

20. Are you on admission/ hospitalization? Yes /No

 a. If Yes, when were you admitted? ………/……/……

21. Have you suffered any respiratory infection(s) recently? Yes / No

 a. If Yes, when was the last time you had a respiratory infection? ………………………………

22. Have you come in contact with a respiratory infected person recently? Yes / No

 a. If Yes, when did it occur? ……………………………………………………………………

23. Have you come in contact with sick or dead bird (s) recently? Yes / No

 a. If Yes, when did it occur? ……………………………………………………………………

24. Do you live alone? Yes / No

 a. If No, how many people do you share your room or live with? ……………………….

25. How many windows are there in your room? …………………………………………...

26a. Do you smoke? Yes / No

 i. If Yes, how long have you been smoking? ……………………………………………..

26b. Do you take in alcohol? Yes / No

 i. If Yes, how long have you been drinking alcohol? ………………………………………

27. Have you been exposed to any form of environmental / passive tobacco smoking recently? Yes/No

 a. If Yes, when was the recent exposure? ………………………………………………………

**Others**

28.Other medical conditions / Diagnosis: ………………...……………………………………..

29. Is patient on any form of antibiotics? Yes /No

 a. If Yes, which type of antibiotics? ……………………………………………………….

## PART III

**Clinical Investigation**

30. Date of ARI symptoms onset: …………………

31. ARI clinical symptoms: fever ≥ 38˚C ☐ cough ☐ rhinorrhea ☐

difficulty in breathing ☐ Sore throat ☐

Others, please specify……………………………………………………………………………..

32.Sample Collection:

| Treatment Day | Date | Fractions taken | Type of Specimen | Result | Remark |
|---|---|---|---|---|---|
| First day | | | | | |
| During | | | | | |
| Last day | | | | | |

Patient's general status……………………………………………………….……………………..
