# Supplementary material for: Occurrence of influenza and bacterial infections in cancer patients receiving radiotherapy in Ghana
Source: PLoS One. 2022 Jul 26;17(7):e0271877. doi: 10.1371/journal.pone.0271877 (PMC9321433; doi:10.1371/journal.pone.0271877)
Supplement: S2 Fig — (PDF) [file pone.0271877.s002.pdf]

ETHICAL CLEARANCE

NOGUCHI MEMORIAL INSTITUTE FOR MEDICAL RESEARCH
Established 1979
A Constituent of the College of Health Sciences
University of Ghana

INSTITUTIONAL REVIEW BOARD

Phone: +233-302-916438 (Direct)
+233-289-522574
Fax: +233-302-502182/513202
E-mail: nirb@noguchi.mimcom.org
Telex No: 2556 UGL GH

Post Office Box LG 581
Legon, Accra
Ghana

My Ref. No: DF.22
Your Ref. No:

4th July, 2018

ETHICAL CLEARANCE

FEDERALWIDE ASSURANCE FWA 00001824 **IRB 00001276**

NMIMR-IRB CPN 091/17-18 **IORG 0000908**

On 4th July 2018, the Noguchi Memorial Institute for Medical Research (NMIMR) Institutional Review Board (IRB) at a full board meeting reviewed and approved your protocol titled:

**TITLE OF PROTOCOL**	:	**Accute Viral Respiratory Infections in Upper-Torso Cancer Patients Undergoing Radiotherapy in Accra, Ghana**
**PRINCIPAL INVESTIGATOR**	:	**Augustina Kwakyewaa. Arjarquah**

Please note that a final review report must be submitted to the Board at the completion of the study. Your research records may be audited at any time during or after the implementation.

Any modification of this research project must be submitted to the IRB for review and approval prior to implementation.

Please report all serious adverse events related to this study to NMIMR-IRB within seven days verbally and fourteen days in writing.

This certificate is valid till 3rd July, 2019. You are to submit annual reports for continuing review.

Signature of Chair: ....
Mrs. Chris Dadzie
(NMIMR – IRB, Chair)

In case of reply the number
And the date of this
Letter should be quoted

My Ref. No. KBTH/[illegible]/[illegible]/[illegible]
Your Ref. No..........................

KORLE BU TEACHING HOSPITAL
P. O. BOX KB 77,
KORLE BU, ACCRA.

Tel: +233 302 667759/673034-6
Fax: +233 302 667759
Email: Info@kbth.gov.gh
pr@kbth.gov.gh
Website: www.kbth.gov.gh

8th November, 2018

AUGUSTINA KWAKYEWAA ARJARQUAH
DEPT. OF BIOCHEMISTRY, CELL AND MOLECULAR BIOLOGY
UNIVERSITY OF GHANA
LEGON

ACUTE VIRAL RESPIRATORY INFECTIONS IN UPPER-TORSO CANCER PATIENTS UNDERGOING RADIOTHERAPY IN ACCRA, GHANA

KBTH-IRB /000116/2018

Investigator: Augustina K. Arjarquah

The Korle Bu Teaching Hospital Institutional Review Board (KBTH IRB) reviewed and granted approval to the study entitled "Acute viral respiratory infections in upper-torso cancer patients undergoing Radiotherapy in Accra, Ghana"

Please note that the Board requires you to submit a final review report on completion of this study to the KBTH-IRB.

Kindly, note that, any modification/amendment to the approved study protocol without approval from KBTH-IRB renders this certificate invalid.

Please report all serious adverse events related to this study to KBTH-IRB within seven days verbally and fourteen days in writing.

This IRB approval is valid till 30th December, 2019. You are to submit annual report for continuing review.

Sincere regards,

MR OKYERE BOATENG
CHAIR (KBTH-IRB)

Cc: The Chief Executive Officer
Korle Bu Teaching Hospital
