# Supplementary material for: Occurrence of influenza and bacterial infections in cancer patients receiving radiotherapy in Ghana
Source: PLoS One. 2022 Jul 26;17(7):e0271877. doi: 10.1371/journal.pone.0271877 (PMC9321433; doi:10.1371/journal.pone.0271877)
Supplement: S1 Table — MLST = Multi-locus sequence type; hlgA = gamma-hemolysin chain II precursor, hlgB = gamma-hemolysin component B precursor, hlgC = gamma-hemolysin component C, luk = leucocidin, luk-PV = Panton Valentine leucocidin; * = nearest hit. (DOCX) [file pone.0271877.s003.docx]

S1 Table. Characterization of S. aureus by spa type, MLST and Exo-toxin genes

| **Isolate** | ***spa* typing** | | **MLST** | **Exo-toxin genes** |
| --- | --- | --- | --- | --- |
|  | ***spa* type** | ***spa* repeat** |  |  |
| **RAC013018-A** | t334 | 11-12-21-17-34-22-25 | **2021** | hlgA & B and lukD & E |
| **RAC033018-B** | Unknown | **-** | **4263908*** | hlgA, B, C and lukE |
| **RAC075018-C** | Unknown | **-** | **580** | hlgA, B, C and lukE |
| **RAC082018-A** | Unknown | **-** | **152** | hlgA & B and lukF-PV & lukS-PV |

MLST = Multi-locus sequence type; hlgA **=** gamma-hemolysin chain II precursor, hlgB = gamma-hemolysin component B precursor, hlgC = gamma-hemolysin component C, luk = leucocidin, luk-PV = Panton Valentine leucocidin; * = nearest hit
